# Supplementary material for: T and NK cell lymphoma cell lines do not rely on ZAP-70 for survival
Source: PLoS One. 2022 Jan 25;17(1):e0261469. doi: 10.1371/journal.pone.0261469 (PMC8789098; doi:10.1371/journal.pone.0261469)
Supplement: S1 Fig — Activation of the TCR signaling pathway was stimulated by the addition of OKT3 10μg/mL over 4h and 18h. Immunoblot analyses was performed on these cell lines and the following proteins detected—phosphorylated and total ZAP70 as well as target substrates of ZAP-70, phosphorylated and total [LAT, SLP76, and p38 MAPK]. (PDF) [file pone.0261469.s001.pdf]

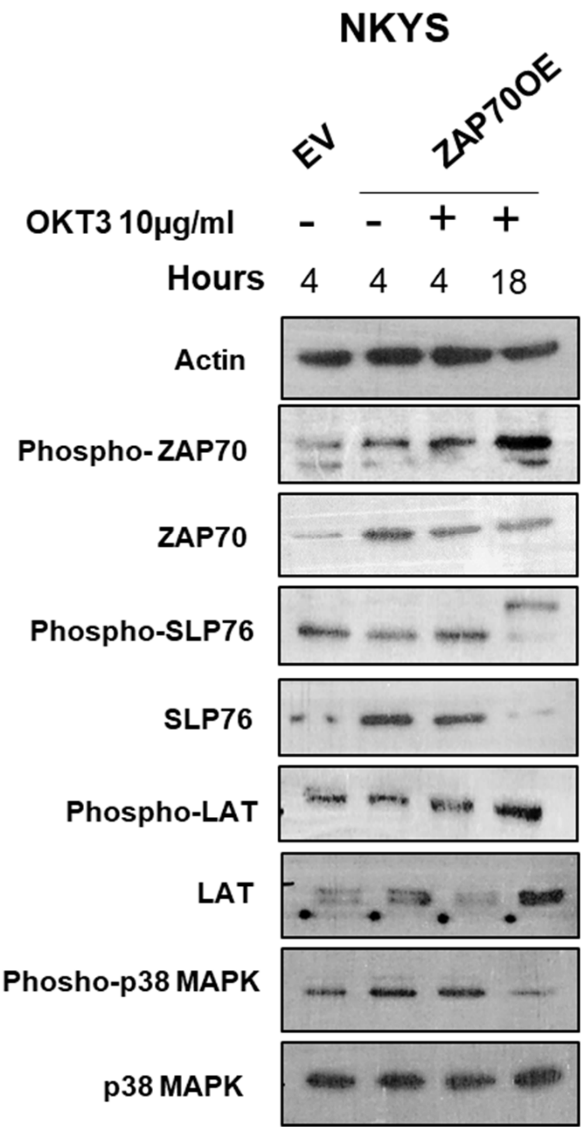

S1 Fig. Addition of OKT3 stimulates an increase in ZAP70 phosphorylation Activation of the TCR signaling pathway was stimulated by the addition of OKT3 10µg/mL over 4h and 18h. Immunoblot analyses was performed on these cell lines and the following proteins detected - phosphorylated and total ZAP70 as well as target substrates of ZAP-70, phosphorylated and total [LAT, SLP76, and p38 MAPK].
