# Supplementary material for: T and NK cell lymphoma cell lines do not rely on ZAP-70 for survival
Source: PLoS One. 2022 Jan 25;17(1):e0261469. doi: 10.1371/journal.pone.0261469 (PMC8789098; doi:10.1371/journal.pone.0261469)
Supplement: S2 Fig — (A) KHYG cells were treated with siNT or siZAP70 and then the effect on the mRNA expression levels of PRDX4, CMYC and ATF5 measured via qRT-PCR. (B) NKYS EV and ZAP70 overexpressing stable cell lines were analysed for levels of mRNA expression of PRDX4, CMYC and ATF5 measured via qRT-PCR. (PDF) [file pone.0261469.s002.pdf]

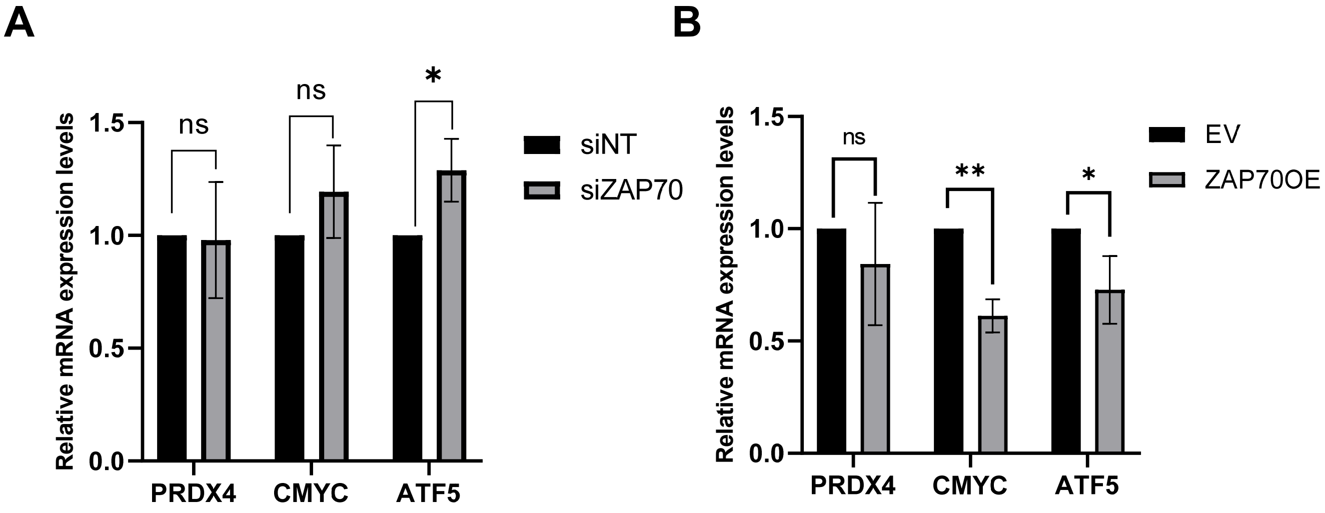


**S2 Fig. E2F and MYC gene targets, PRDX4, CMYC and ATF5 are not significantly downregulated upon ZAP70 knockdown.** (A) KHYG cells were treated with siNT or siZAP70 and then the effect on the mRNA expression levels of PRDX4, CMYC and ATF5 measured via qRT-PCR. (B) NKYS EV and NKYS ZAP70 overexpressing stable cell lines were analysed for levels of mRNA expression of PRDX4, CMYC and ATF5 measured via qRT-PCR
