# Supplementary material for: T and NK cell lymphoma cell lines do not rely on ZAP-70 for survival
Source: PLoS One. 2022 Jan 25;17(1):e0261469. doi: 10.1371/journal.pone.0261469 (PMC8789098; doi:10.1371/journal.pone.0261469)
Supplement: S3 Fig — KHYG and HANK-1 cell lines which have been electroporated with either siNT or siZAP70 were evaluated by a cell viability assay, Cell-Titer Glo (Promega) after 48h treatment with different doses of Gefitinib. (PDF) [file pone.0261469.s003.pdf]

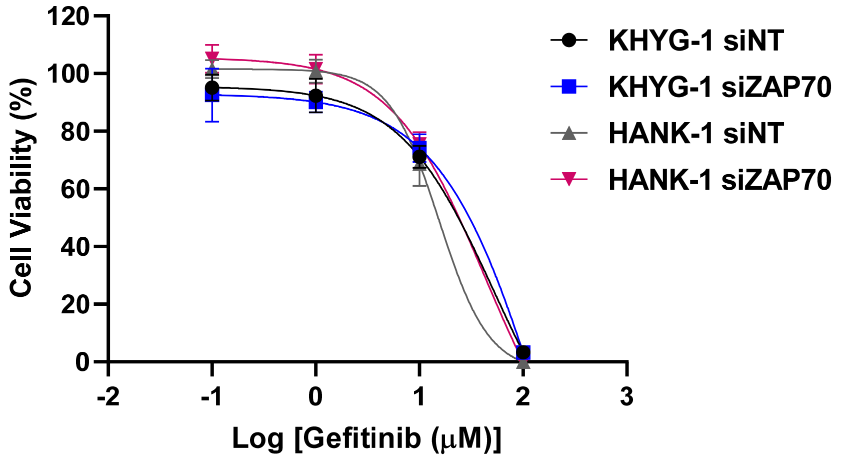

S3 Fig. Depletion of ZAP70 in NKTL cell lines do not confer any sensitivity to Gefitinib treatment KHYG and HANK-1 cell lines which have been electroporated wit either siNT or siZAP70 were evaluated by a cell viability assay, Cell-Titer Glo (Promega) after 48h treatment with different doses of Gefitinib.
