# Supplementary material for: T and NK cell lymphoma cell lines do not rely on ZAP-70 for survival
Source: PLoS One. 2022 Jan 25;17(1):e0261469. doi: 10.1371/journal.pone.0261469 (PMC8789098; doi:10.1371/journal.pone.0261469)

Supplementary 1_Raw Images – Figure 1

Main Figure reference – Figure 1A, 1B

1A

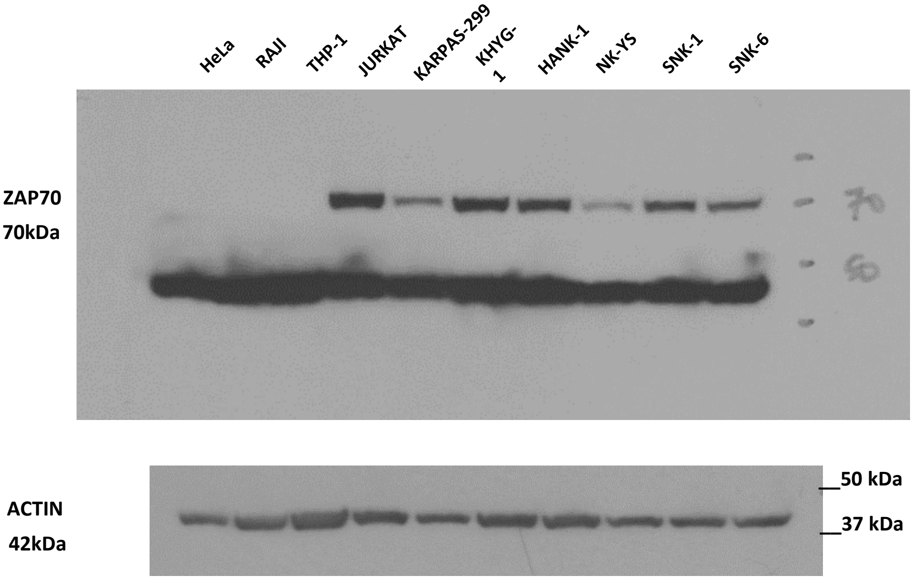

1B

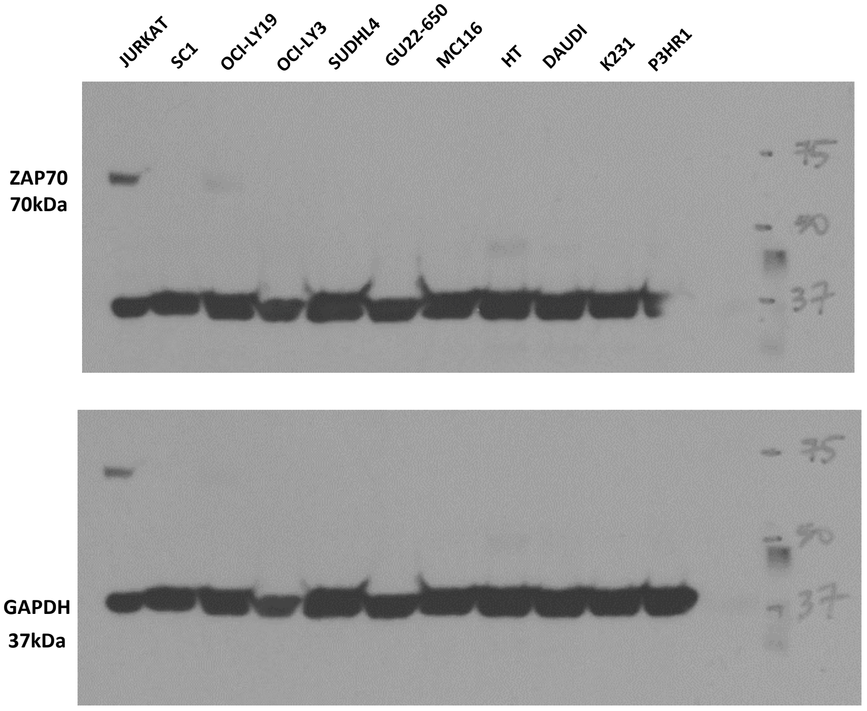

Supplementary 1 _Raw Images – Figure 2

Main Figure reference – Figure 2A

2A

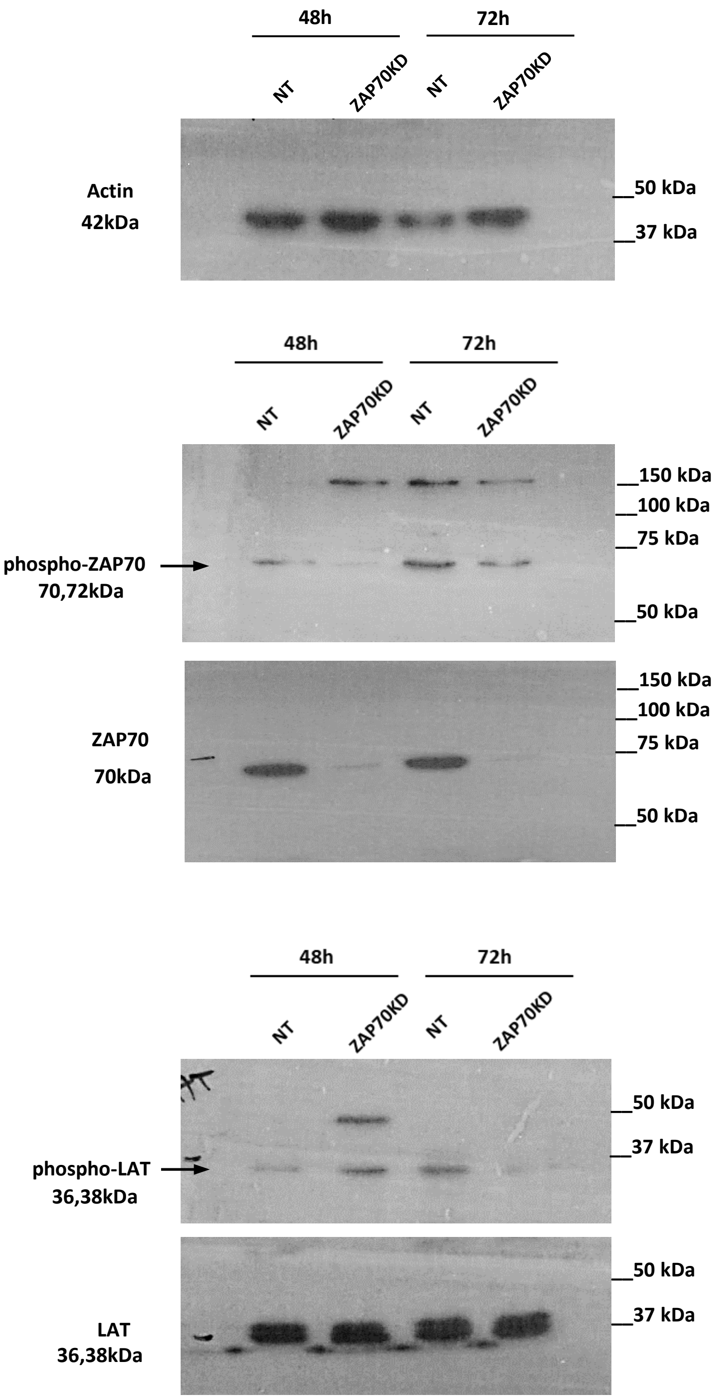

Supplementary 1 _Raw Images – Figure 2

Main Figure reference – Figure 2A continued

2A

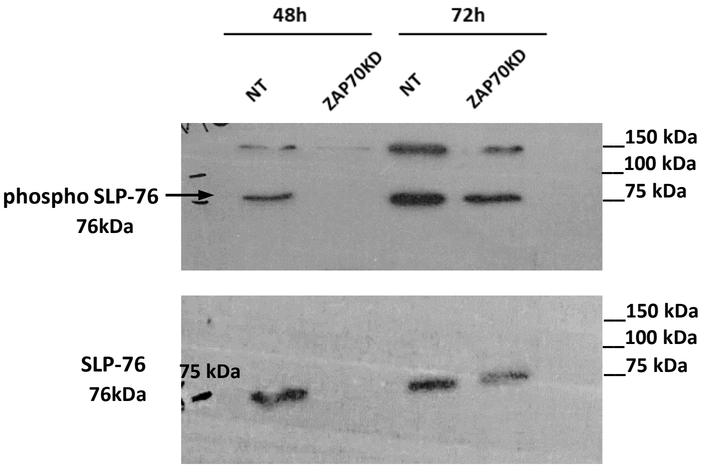

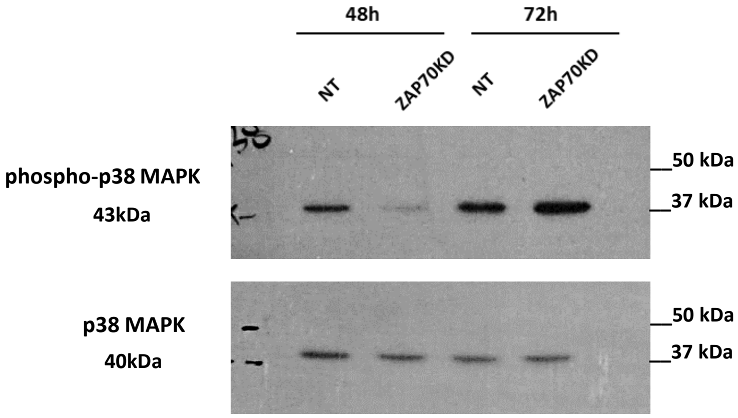

Supplementary 1 _Raw Images – Figure 3

Main Figure reference – Figure 2C

2C

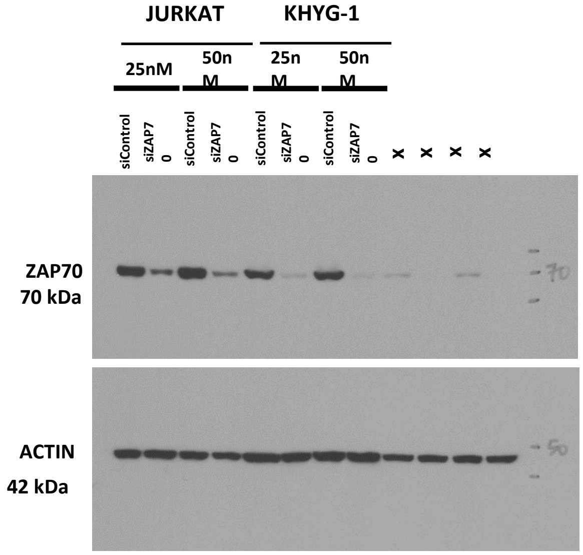

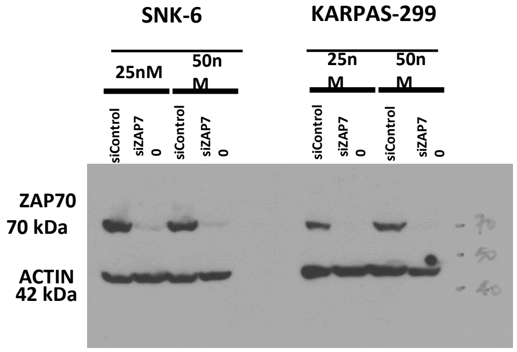

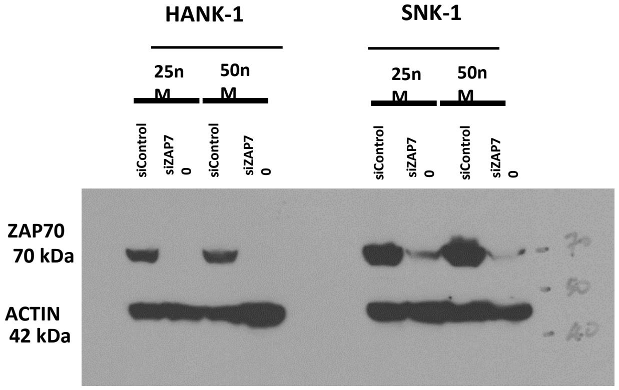

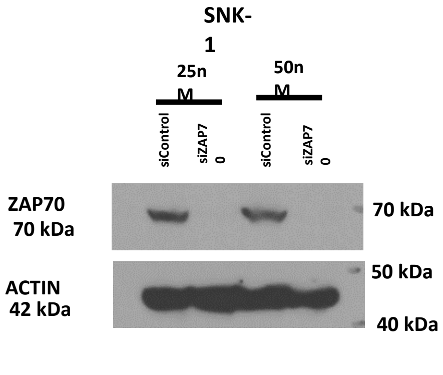

Supplementary 1 _Raw Images – Figure 3

Main Figure reference – Figure 2C continued

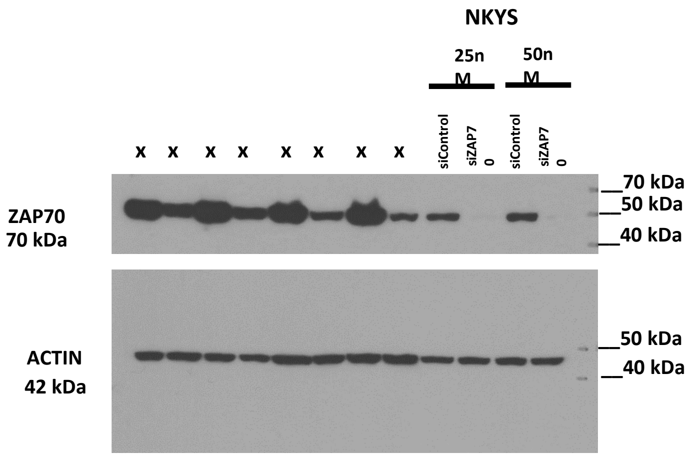

Supplementary 1 _Raw Images – Figure 4

Main Figure reference – Figure 4A

4A

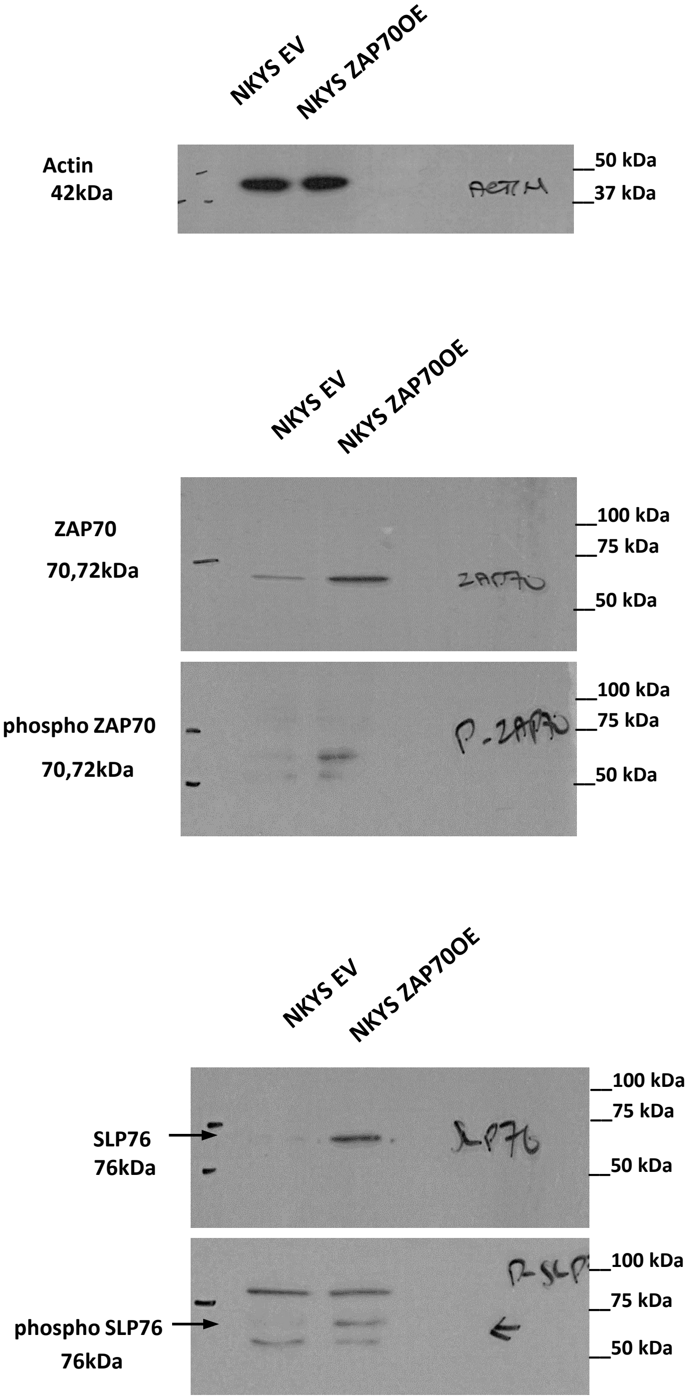

Supplementary 1 _Raw Images – Figure 4

Main Figure reference – Figure 4A continued

4A

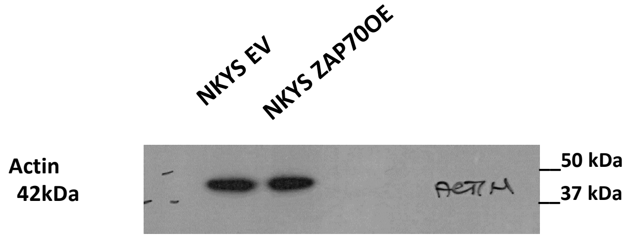

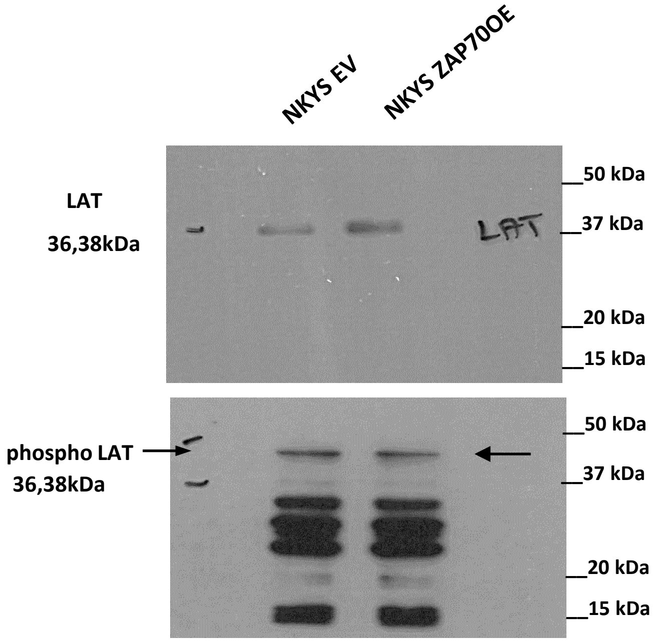

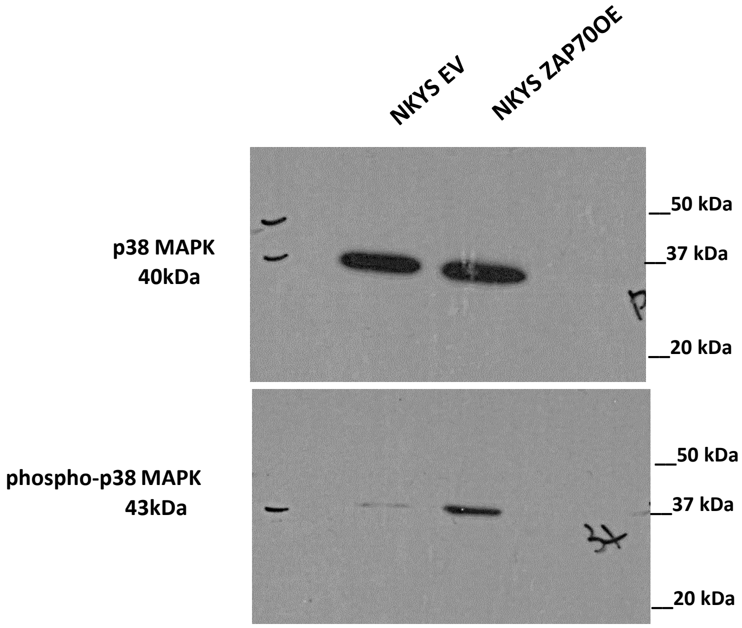

Supplementary 1 _Raw Images – Figure 5

Main Figure reference – Supplementary Figure 1

Supplementary Figure 1

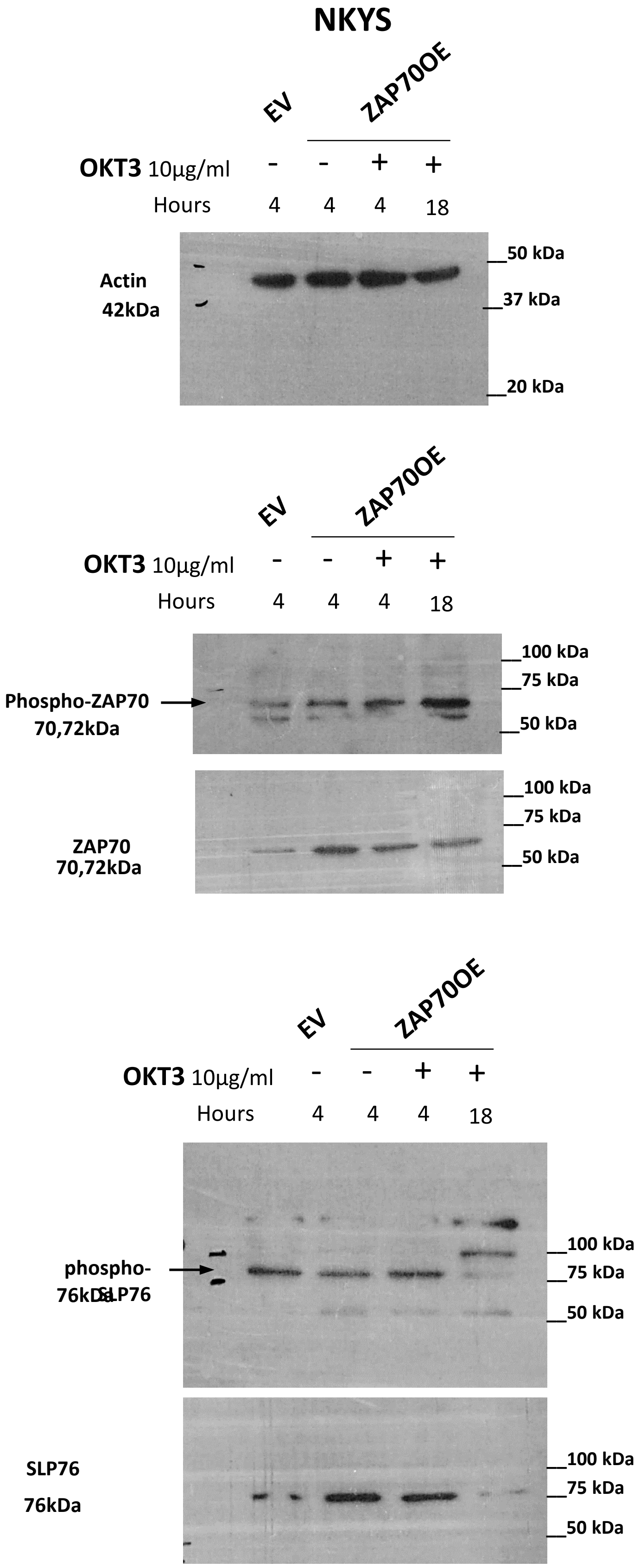

Supplementary 1 _Raw Images – Figure 5

Main Figure reference – Supplementary Figure 1 continued

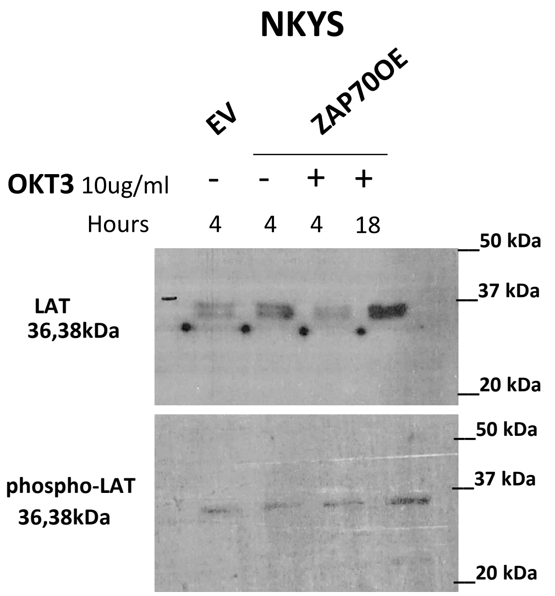

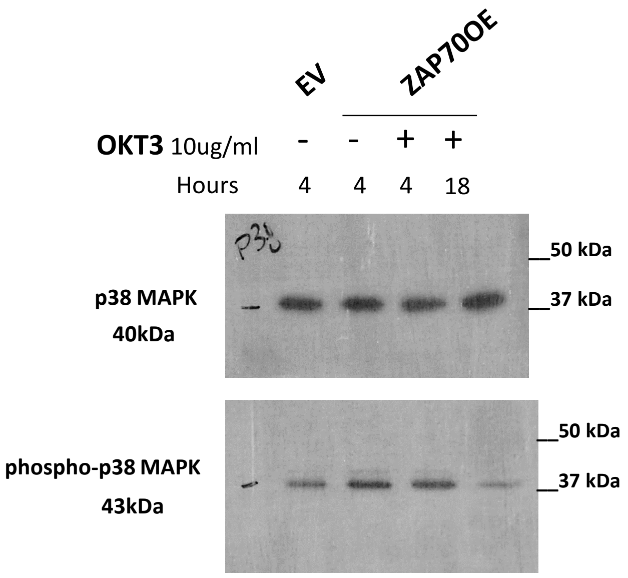

Supplement: S1 Raw images — (PDF) [file pone.0261469.s004.pdf]
