# Supplementary material for: T and NK cell lymphoma cell lines do not rely on ZAP-70 for survival
Source: PLoS One. 2022 Jan 25;17(1):e0261469. doi: 10.1371/journal.pone.0261469 (PMC8789098; doi:10.1371/journal.pone.0261469)
Supplement: Attachment — Submitted filename: Response to Reviewers.docx [file pone.0261469.s005.docx]

| Editor’s comments | Author’s response |
| --- | --- |
| The authors need to address evidence that reduced and over expression of ZAP-70 affects T cell receptor signalling and cell death. A time course following T cell receptor activation should be shown. Finally RNA-seq analysis needs to be further explained. | We have confirmed that siRNA knockdown of ZAP-70 suppresses the phosphorylation of ZAP70 target substrates in the TCR signalling pathway - phospho-SLP76 and phospho-p38MAPK are downregulated at 48h and phosoho-LAT at 72h (Figure 2A). We also observe some restoration of the phosphorylation levels of p38 and SLP-76 at 72h despite continued ZAP-70 knockdown. Taken together with the rest of our data, this suggests that T/NK lymphoma cell lines may have a mechanism to compensate for the loss of ZAP-70 signalling. This is further discussed in the manuscript.  Conversely, overexpression of ZAP-70 results in the increased expression and phosphorylation of ZAP-70 substrates SLP-76, LAT and p38 MAPK (Figure 4A).  This is further confirmed by a time course activation of the NKTL cells with OKT3 corresponding with increased activation and phosphorylation of ZAP-70 and LAT (Supplementary Fig 1). The TCR-mediated phosphorylation and activity of ZAP-70 is also reported to mediate negative feedback mechanisms which may account for the decrease in p38 and pSLP76 phosphorylation at 18h.  We also confirmed that the loss and gain of ZAP-70 mediated signalling in the above stated NKTL models did not result in any change to cell viability or apoptosis.  Finally, for the RNA seq analysis, we have reanalysed the data set and updated the corresponding gene set enrichment plots as well as volcano plots. We have also included Tables 1 and 2 to present relevant outcomes of the analysis. 1. (Fig 3A., Table 1) We have reanalysed the RNA-seq to include gene set enrichment for C2- curated gene sets. This is important so that we can study the effect of ZAP70 knockdown on TCR associated pathways particularly downstream signalling and gene targets. This analysis demonstrates that ZAP70 knockdown has no significant effect on TCR associated pathways and suggests that the role of ZAP70 in NKTL may be redundant. |
| Reviewer Comments | Authors’ Response |
| 1. There is no experimental evidence that reduced and over expression of ZAP-70 affects T cell receptor signalling. This could affect the interpretation of the results. | Response as above (Figure 2A, 4A) |
| 2.The use of siRNA showed reduced ZAP-70 expression failed to show a time course of knockdown and level of apoptosis. | We now show knockdown at 48h and 72h and confirm that there is no change in the levels of apoptosis induced over time (Figure 2B) |
| 3. Need to induce apoptosis in these cells to determine if the ability of these cells to undergo cell death. | Apoptosis was successfully induced in NKTL cell lines by treatment with Obatoclax, a BH3 mimetic (Figure 2B, 2E) |
| 4. For the gefitinib experiment, knockdown of ZAP70 and the effects of gefitinib treatment should be investigated | Both the knockdown and overexpression model of ZAP-70 do not show any differential response to Gefitinib. (Figure 5B, Supplementary figure 3) |
| 5. RNAseq shows affects in cell cycle (Myc or E2F targets) but nucleofector is very stressful on cells. This could alter cellular functions. Should be validated by over expression of ZAP-70. Should be evaluated following TCR signalling as well. | MYC and E2F targets gene sets were enriched in the Hallmark analysis of siZAP-70 over si NT control – si NT control are cells which underwent NEON transfection with a non-targeting control. Thus, any stress/changes in gene expression induced by the transfection process in siZAP70 would also show up in the si control. Thus, the negative enrichment for MYC and E2F targets are likely independent of that.  We identified and selected genes that were top ranked in the gene set list for MYC and E2F targets – PRDX4, CMYC and ATF5. PRDX4 and MYC were not significantly downregulated by the knockdown while ATF5 was just slightly upregulated contrary to what is expected. The same genes studied in the OE cell lines demonstrate either no significant change or a slight downregulation. Nonetheless, the change in gene expression levels are less than 1.5 fold and may not have a significant impact on the survival and death signalling pathways as observed and confirmed by our functional cell viability and apoptosis assays.  In order to study the RNAseq data in light of the effect of ZAP-70 on TCR signalling downstream, the gene set enrichment analysis was repeated with a different gene set parameter C2(Broad Institute).  The analyses showed that there was no significant enrichment for TCR signalling pathways in all 3 gene sets. Furthermore, besides HANK-1 , the other 2 cell lines showed minimal or no significant gene set enrichment in ZAP-70 KD cells. This suggests yet again that ZAP-70 may be largely redundant in NKTL. |
| 6. No functional validation of the results in RNA sequencing. | The expression of several genes (AHNAK2, PMEPA1, KLHDC7B, ZAP70) were validated in the ZAP70 KD model of KHYG. |
| 7.The downstream signalling that was affected on Zap-70 knockdown studied by RNA-seq analysis needs to be further explained. Potential hypothesis for pathways that may be causing Zap-70 independent TCR signalling can also be better explained in discussion. Immune dysregulation and whether PD1/PDL1 expression that was altered on Zap-70 manipulation in these cell lines if analysed should be reported. | As described in the reply to point 5, we repeated the GSEA. The cell line HANK1 has a small number of genes differentially expressed while the other two cell lines have no significant differential gene expression.  We also confirmed that there are no differentially expressed genes related to the TCR signalling pathway between ZAP 70 KD and WT cells.  We have not studied PD1/PDL1 expression in the cell lines at the protein level. However there was no difference in the expression of these genes on GSEA.  We have included three hypotheses for ZAP-70 independent TCR signalling in the discussion. |
